# Recall urticaria caused by vedolizumab: case report and literature review

**DOI:** 10.3389/falgy.2026.1781121

**Published:** 2026-04-21

**Authors:** Mio Kozuma, Reiko Hara, Yumiko Sakuragi, Natsuko Saito-Sasaki, Yu Sawada

**Affiliations:** Department of Dermatology, University of Occupational and Environmental Health, Kitakyushu City, Japan

**Keywords:** case report, drug eruption, literature review, recall urticaria, vedolizumab

## Abstract

Recall urticaria is a rare hypersensitivity phenomenon characterized by the reappearance of urticarial wheals strictly confined to previously exposed skin sites following systemic re-exposure to a trigger. Although reported with several drugs, its clinical features and underlying mechanisms remain poorly defined. A 25-year-old man with ulcerative colitis developed acute pruritic wheals localized exclusively to prior subcutaneous injection sites approximately 15 min after intravenous administration of vedolizumab. He had experienced repeated localized injection-site reactions during prior subcutaneous therapy. Histopathology revealed mild perivascular inflammation with eosinophils. The eruption resolved spontaneously within 24 h without systemic symptoms, and vedolizumab therapy was continued. This case represents the first report of vedolizumab-associated recall urticaria. A review of previously reported cases highlights strict site specificity as the defining feature, irrespective of the route or timing of re-exposure. Recall urticaria should be recognized as a site-specific hypersensitivity reaction distinct from systemic drug allergy.

## Introduction

Recall reactions confined to previously exposed skin sites represent a rare but distinctive hypersensitivity phenomenon following re-exposure to an antigen or drug (1). Among these, recall urticaria is characterized by the reappearance of wheals strictly limited to prior injection or exposure sites. However, the number of reported cases remains extremely limited, and most descriptions are confined to single case reports (1, 2). As a result, the clinical spectrum of recall urticaria and its association with newer biologic therapies remain incompletely defined. Vedolizumab is a gut-selective anti-α4β7 integrin monoclonal antibody, which is widely used for inflammatory bowel disease (3). To date, recall urticaria associated with vedolizumab has not been reported. Here, we describe a patient who developed wheals strictly confined to previous subcutaneous injection sites shortly after intravenous re-exposure to vedolizumab. We also review the previously reported cases of recall urticaria due to drugs.

## Case presentation

A 25-year-old Japanese man with a 2-year history of ulcerative colitis was referred to our dermatology department for evaluation of acute pruritic wheals confined to the bilateral abdomen. He had started vedolizumab therapy (300 mg intravenously) for induction and maintenance approximately 2 years and 2 months before presentation. Approximately 5 months before his first dermatology visit, the regimen was switched to subcutaneous vedolizumab (108 mg). During the subcutaneous phase, he repeatedly experienced localized injection-site reactions characterized by induration and pruritus at the abdominal injection sites. The patient had no history of treatment with other monoclonal antibodies prior to vedolizumab therapy, making cross-reactivity with other biologics unlikely.

On the day of presentation, vedolizumab was switched back to intravenous administration (300 mg). Approximately 15 min after the infusion, he developed intense pruritus followed by well-demarcated urticarial wheals strictly confined to the bilateral abdominal areas corresponding to the previous subcutaneous injection sites. Notably, similar site-specific urticarial eruptions recurred reproducibly after subsequent intravenous administrations of vedolizumab, without involvement of other skin areas or systemic symptoms. He was therefore referred to our department for further evaluation.

**Figure 1:**
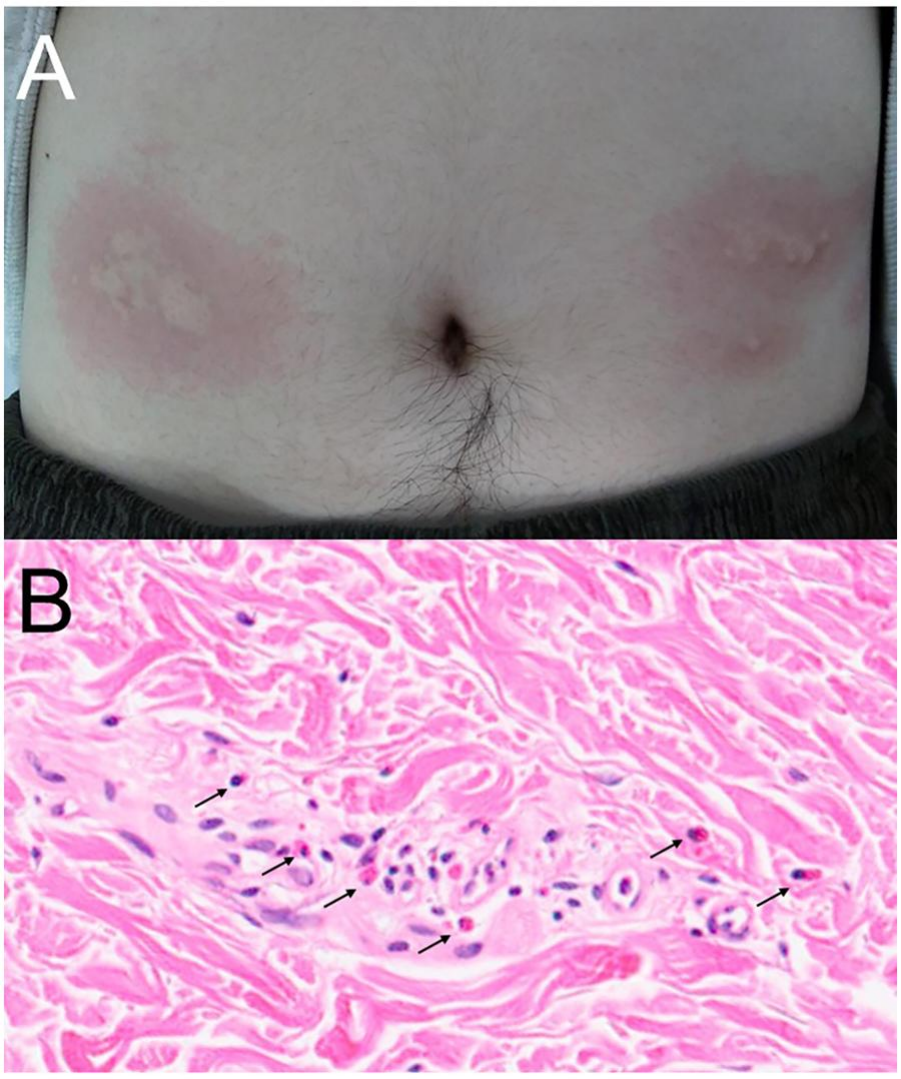
Clinical manifestation and histological examination. **(A)** Well-demarcated erythematous wheals symmetrically localized to previous subcutaneous injection sites on the abdomen, appearing shortly after intravenous vedolizumab administration. **(B)** Histopathological examination showing mild perivascular inflammatory cell infiltration with eosinophils (arrows) in the dermis without features of delayed-type hypersensitivity (hematoxylin and eosin stain).

On physical examination, edematous wheals with erythematous borders strictly confined to the former injection sites (Figure 1A). A skin biopsy obtained from an affected site demonstrated mild perivascular inflammatory cell infiltration with scattered eosinophils in the superficial dermis (Figure 1B). The eruption resolved spontaneously within 24 h without systemic treatment and without residual pigmentation. Based on the rapid onset, strict site-specific localization, and transient clinical course, the clinical findings were most consistent with an immediate-type recall reaction occurring at previous injection sites following systemic re-exposure to vedolizumab. Because the reaction was limited to the skin without systemic involvement, vedolizumab therapy was continued without modification. Similar reactions recurred after each subsequent intravenous administration, with no changes in the dosing interval. The intensity and duration of the eruptions remained consistent across episodes, and no antihistamine premedication was administered.

## Discussion

Recall urticaria has been reported only sporadically, mostly as isolated case reports involving heterogeneous triggers and routes of re-exposure (1, 2). Despite this variability, a consistent clinical feature across reported cases is the strict localization of wheals to previously exposed skin, which distinguishes recall urticaria from generalized drug-induced urticaria or infusion-related reactions.

The present case represents a clear example of immediate-type drug-induced recall urticaria. Urticarial wheals developed within 15 min of intravenous vedolizumab administration and were strictly confined to prior subcutaneous injection sites, resolving spontaneously within 24 h without systemic involvement. To our knowledge, vedolizumab has not previously been reported as a trigger of recall urticaria and is therefore not included among earlier cases summarized in Table 1. The rapid onset and urticarial morphology argue strongly against T cell–mediated delayed hypersensitivity and are inconsistent with conventional injection-site reactions, which typically occur only at the site of drug administration.

**Table 1:** Reported cases of recall urticaria triggered by drugs.

First author	Gender/age	Trigger/agent	Route of re-exposure	Site of recall	Onset
Kelso JM, et al. (10)	26/Male	Immunotherapy	Intradermal injection	Previous injection sites	N.D
Yedidi RS, et al. (11)	20/Female	Agalsidase	Intravenous injection	Infusion site	30 min
Karaayvaz M, et al. (1)	11/Male	Immunotherapy	Intradermal injection	Previous injection sites	48–72 h
Rinn K, et al. (13)	48/Female	HER-2/neu peptide vaccine	Intradermal injection	Whole body	5 min
Caliskaner Z, et al. (14)	30/Female	Heparin	Intradermal injection	Previous injection sites	N.D
Weber HO, et al. (15)	42/Female	Dalteparin	Intradermal injection	Previous injection sites	1 h
Tan C, et al. (2)	31/Male	Norfloxacin	Oral	Previous levofloxacin IV site	1 h
Li PH, et al. (16)	29/Female	Adalimumab	Intradermal injection	Previous injection sites	1 h
Cimbollek S, et al. (17)	30/Female	Ibuprofen	Oral	Previous house dust mice immunotherapy injection sites	40 min
De Aramburu Mera T, et al. (18)	29/female	Anakinra	Subcutaneous injection	Previous injection sites	60 min
Ta V, et al. (19)	27/Female	Immunotherapy	Subcutaneous injection	Previous injection sites	N.D
Our case	25/Male	Vedolizumab	Intradermal injection	Previous injection sites	15 min

N.D: Not Described.

(Kelso, 1994 #15) (Weber, 2009 #25).

Although the pathophysiology of recall urticaria remains incompletely understood, available evidence supports a central role for mast cell–mediated mechanisms in immediate-type reactions. Mast cells are long-lived tissue-resident immune cells that persist within the skin and serve as primary effector cells in urticaria (4). Vedolizumab selectively targets the α4β7 integrin, which plays a critical role in lymphocyte homing to the gastrointestinal tract. While α4β7 integrin is primarily associated with lymphocyte trafficking (5), integrin-mediated pathways are also involved in the localization and retention of other immune cells, including mast cells, within tissues. Therefore, modulation of α4β7-related pathways by vedolizumab may influence the local immune environment beyond the gastrointestinal tract. In the present case, prior local exposure through subcutaneous injections may have established a population of primed mast cells at specific skin sites, which were subsequently reactivated upon systemic re-exposure, leading to localized wheal formation. Experimental studies suggest that mast cells can exhibit sustained, context-dependent alterations in functional responsiveness following prior inflammatory stimulation, even in the absence of antigen-specific adaptive immune memory (6). Such properties may provide a plausible explanation for the site-specific reactivation observed in recall urticaria without invoking classical immune memory.

In the present case, eosinophils were observed histologically within lesional skin. Although eosinophils are not considered primary initiators of urticaria, they are known to reside constitutively within peripheral tissues, including the skin, and can respond rapidly to local inflammatory cues (7, 8). Following mast cell activation, mediators such as histamine, lipid mediators, and chemokines may promptly activate tissue-resident eosinophils, which can act as secondary amplifiers of vascular permeability and pruritus. Thus, eosinophil involvement in this case is best interpreted as contributing to the amplification of localized wheal formation rather than serving as a primary memory-bearing or initiating effector cell.

Tissue-resident memory T cells have been implicated in other forms of site-specific immune recall in the skin (9); however, they are unlikely to function as primary effector cells in immediate urticarial responses such as the present case. Their potential contribution, if any, would more plausibly involve modulation of the local inflammatory milieu rather than direct induction of wheal formation.

Based on the published cases summarized in Table 1, several shared clinical characteristics of recall urticaria can be identified (2, 10–19). First, in the majority of reported cases, recall urticaria occurred at sites that had been previously exposed to the causative agent via local administration, most commonly through intradermal or subcutaneous injection. Even in cases in which re-exposure occurred via a different route, such as oral administration, prior local exposure through injection was documented, supporting the concept that local sensitization is a prerequisite for recall reactions.

Second, although the onset of recall urticaria is often rapid, occurring within minutes to approximately one hour after re-exposure in many cases, several reports describe a delayed onset of up to several days. Importantly, these delayed presentations have nevertheless been classified as recall urticaria in the literature, indicating that strict temporal criteria are not essential for diagnosis. Rather, the defining feature across reports is the reproducible localization of wheals to previously exposed skin sites.

Regarding triggering agents, recall urticaria has been reported with a wide range of substances, including protein-based therapeutics such as allergen immunotherapy extracts, monoclonal antibodies, cytokines, and peptide vaccines, as well as low-molecular-weight compounds such as antibiotics and nonsteroidal anti-inflammatory drugs. Notably, however, many cases involve agents that are administered repeatedly and locally, particularly by subcutaneous or intradermal injection, suggesting that repeated local exposure may facilitate the development of site-specific hypersensitivity.

With respect to patient characteristics, no clear sex predilection is evident from the limited number of reported cases. Among the cases summarized in Table 1, females appear to be slightly more frequent than males, approximately in a 2:1 ratio; however, the small sample size and heterogeneity of reports preclude any definitive conclusions. Patient ages span a broad range, indicating that recall urticaria is not restricted to a specific age group.

Skin tests or eosinophil activation tests were not performed in this case, which limits definitive confirmation of vedolizumab as the causative agent. However, the reproducibility of site-specific reactions following repeated administrations strongly supports a causal relationship.

Taken together, these observations support the interpretation of recall urticaria as a site-specific hypersensitivity phenomenon associated with prior local exposure, rather than a conventional systemic drug reaction. The reproducible localization of wheals to previously exposed skin sites, irrespective of the route or timing of re-exposure, remains the most consistent and distinguishing clinical feature.

## Data Availability

The original contributions presented in the study are included in the article/Supplementary Material, further inquiries can be directed to the corresponding author.

## References

[1] Karaayvaz M Ozangüç N. Recall urticaria: a case report. J Allergy Clin Immunol. (1996) 97(6):1419–20. 10.1016/S0091-6749(96)70215-4 8648043

[2] Tan C Zhu WY Min ZS. Recall urticaria related to levofloxacin. J Eur Acad Dermatol Venereol. (2008) 22(5):616–7. 10.1111/j.1468-3083.2007.02412.x 18410619

[3] Feagan BG Rutgeerts P Sands BE Hanauer S Colombel JF Sandborn WJ Vedolizumab as induction and maintenance therapy for ulcerative colitis. N Engl J Med. (2013) 369(8):699–710. 10.1056/NEJMoa1215734 23964932

[4] Galli SJ Kalesnikoff J Grimbaldeston MA Piliponsky AM Williams CM Tsai M. Mast cells as “tunable” effector and immunoregulatory cells: recent advances. Annu Rev Immunol. (2005) 23:749–86. 10.1146/annurev.immunol.21.120601.141025 15771585

[5] Park EJ Mora JR Carman CV Chen J Sasaki Y Cheng G Aberrant activation of integrin alpha4beta7 suppresses lymphocyte migration to the gut. J Clin Invest. (2007) 117(9):2526–38. 10.1172/JCI31570 17786243 PMC1952632

[6] De Zuani M Dal Secco C Tonon S Arzese A Pucillo CEM Frossi B. LPS guides distinct patterns of training and tolerance in mast cells. Front Immunol. (2022) 13:835348. 10.3389/fimmu.2022.835348 35251027 PMC8891506

[7] Chojnacki A Wojcik K Petri B Aulakh G Jacobsen EA LeSuer WE Intravital imaging allows real-time characterization of tissue resident eosinophils. Commun Biol. (2019) 2:181. 10.1038/s42003-019-0425-3 31098414 PMC6513871

[8] Gurtner A Crepaz D Arnold IC. Emerging functions of tissue-resident eosinophils. J Exp Med. (2023) 220(7):e20221435. 10.1084/jem.20221435 37326974 PMC10276195

[9] Clark RA. Resident memory T cells in human health and disease. Sci Transl Med. (2015) 7(269):269rv1. 10.1126/scitranslmed.3010641 25568072 PMC4425129

[10] Kelso JM Hugh MY Lin FL. Recall urticaria. J Allergy Clin Immunol. (1994) 93(5):949–50. 10.1016/0091-6749(94)90392-1 8182240

[11] Yedidi RS Bernstein JA. Recall urticaria with infusion of agalsidase beta. Ann Allergy Asthma Immunol. (2023) 131(5):672–4. 10.1016/j.anai.2023.08.001 37557952

[12] Karaayvaz M Ozangüç N. Late recall urticaria. J Investig Allergol Clin Immunol. (1998) 8(5):309–11. 9827429

[13] Rinn K Schiffman K Otero HO Disis ML. Antigen-specific recall urticaria to a peptide-based vaccine. J Allergy Clin Immunol. (1999) 104(1):240–2. 10.1016/S0091-6749(99)70143-0 10400869

[14] Caliskaner Z Karaayvaz M Oztürk S. Recurrent urticaria lesions in a heparin-allergic patient: most likely another form of “recall urticaria”. J Investig Allergol Clin Immunol. (2005) 15(1):78–80. 15864888

[15] Weber HO Fischer J Kneilling M Caroli U Rocken M Biedermann T. Recall urticaria induced by skin tests with heparin. Br J Dermatol. (2009) 161(1):187–9. 10.1111/j.1365-2133.2009.09150.x 19438465

[16] Li PH Watts TJ Lui MS Lau CS Chung HY. Recall Urticaria in Adalimumab hypersensitivity. J Allergy Clin Immunol Pract. (2018) 6(3):1032–3. 10.1016/j.jaip.2017.10.031 29175008

[17] Cimbollek S Ávila-Castellano MR Labella M Baynova K Aramburu T Quiralte J. Recall Urticaria: aspirin also induces it. J Investig Allergol Clin Immunol. (2018) 28(2):131–2. 10.18176/jiaci.0219 29661741

[18] De Aramburu Mera T Reguero Capilla M Ochando Diez-Canseco M Prados Castaño M. Recall injection-site reactions after treatment with anakinra. J Investig Allergol Clin Immunol. (2021) 31(3):275–6. 10.18176/jiaci.0642 34155980

[19] Ta V White AA. An unusual case of recurrent “recall urticaria” in a patient on immunotherapy. J Allergy Clin Immunol Pract. (2014) 2(4):459–60. 10.1016/j.jaip.2014.03.003 25017536

